# Relative Wash-In Rate in Dynamic Contrast-Enhanced Magnetic Resonance Imaging as a New Prognostic Biomarker for Event-Free Survival in 82 Patients with Osteosarcoma: A Multicenter Study

**DOI:** 10.3390/cancers16111954

**Published:** 2024-05-21

**Authors:** Gijsbert M. Kalisvaart, Richard E. Evenhuis, Willem Grootjans, Thomas Van Den Berghe, Martijn Callens, Judith V. M. G. Bovée, David Creytens, Hans Gelderblom, Frank M. Speetjens, Lore Lapeire, Gwen Sys, Marta Fiocco, Koenraad L. Verstraete, Michiel A. J. van de Sande, Johan L. Bloem

**Affiliations:** 1 Department of Radiology, Leiden University Medical Center, 2333 Leiden, The Netherlands; g.m.kalisvaart@lumc.nl (G.M.K.); w.grootjans@lumc.nl (W.G.); j.l.bloem@lumc.nl (J.L.B.); 2 Department of Orthopedic Surgery, Leiden University Medical Center, Albinusdreef 2, 2333 Leiden, The Netherlands; m.a.j.van_de_sande@lumc.nl; 3 Department of Radiology, Ghent University Hospital, 9000 Ghent, Belgium; thovdnbe.vandenberghe@ugent.be (T.V.D.B.); martijn.callens@ugent.be (M.C.); koenraad.verstraete@ugent.be (K.L.V.); 4 Department of Pathology, Leiden University Medical Center, 2333 Leiden, The Netherlands; j.v.m.g.bovee@lumc.nl; 5 Department of Pathology, Ghent University Hospital, 9000 Ghent, Belgium; david.creytens@uzgent.be; 6 Department of Medical Oncology, Leiden University Medical Center, 2333 Leiden, The Netherlands; a.j.gelderblom@lumc.nl (H.G.); f.m.speetjens@lumc.nl (F.M.S.); 7 Department of Medical Oncology, Ghent University Hospital, 9000 Ghent, Belgium; lore.lapeire@uzgent.be; 8 Department of Orthopedic Surgery and Traumatology, Ghent University Hospital, 9000 Ghent, Belgium; gwen.sys@uzgent.be; 9 Department of Biomedical Science, Section Medical Statistics, Leiden University Medical Center, 2333 Leiden, The Netherlands; m.fiocco@math.leidenuniv.nl; 10 Center for Pediatric Oncology, Princess Maxima Center, 3584 Utrecht, The Netherlands; 11 Mathematical Institute, Leiden University, 2300 Leiden, The Netherlands

**Keywords:** osteosarcoma, neoadjuvant chemotherapy, response monitoring, histological response, dynamic contrast-enhanced MRI, survival outcome

## Abstract

**Simple Summary:**

This study explores the potential of the relative wash-in rate (rWIR) in dynamic contrast-enhanced MRI as a prognostic factor for event-free survival (EFS) in osteosarcoma patients. Eighty-two patients were retrospectively included, and rWIR was determined based on preoperative imaging. Patients with rWIR < 2.3 were considered to have a poor radiological response, while those with rWIR ≥ 2.3 had a good response. This study identified that poor radiological response (rWIR < 2.3) was associated with shorter EFS, even when adjusted for traditional prognostic factors. The 2- and 5-year EFS rates for patients with rWIR ≥ 2.3 were 85% and 75%, compared to 55% and 50% for those with rWIR < 2.3. The findings suggest that the predicted poor chemo response with MRI is associated with shorter EFS and shows similar results to histological response evaluation. rWIR is a potential tool for future response-based individualized healthcare in osteosarcoma patients.

**Abstract:**

Background: The decreased perfusion of osteosarcoma in dynamic contrast-enhanced (DCE) MRI, reflecting a good histological response to neoadjuvant chemotherapy, has been described. Purpose: In this study, we aim to explore the potential of the relative wash-in rate as a prognostic factor for event-free survival (EFS). Methods: Skeletal high-grade osteosarcoma patients, treated in two tertiary referral centers between 2005 and 2022, were retrospectively included. The relative wash-in rate (rWIR) was determined with DCE-MRI before, after, or during the second cycle of chemotherapy (pre-resection). A previously determined cut-off was used to categorize patients, where rWIR < 2.3 was considered poor and rWIR ≥ 2.3 a good radiological response. EFS was defined as the time from resection to the first event: local recurrence, new metastases, or tumor-related death. EFS was estimated using Kaplan–Meier’s methodology. Multivariate Cox proportional hazard model was used to estimate the effect of histological response and rWIR on EFS, adjusted for traditional prognostic factors. Results: Eighty-two patients (median age: 17 years; IQR: 14–28) were included. The median follow-up duration was 11.8 years (95% CI: 11.0–12.7). During follow-up, 33 events occurred. Poor histological response was not significantly associated with EFS (HR: 1.8; 95% CI: 0.9–3.8), whereas a poor radiological response was associated with a worse EFS (HR: 2.4; 95% CI: 1.1–5.0). In a subpopulation without initial metastases, the binary assessment of rWIR approached statistical significance (HR: 2.3; 95% CI: 1.0–5.2), whereas its continuous evaluation demonstrated a significant association between higher rWIR and improved EFS (HR: 0.7; 95% CI: 0.5–0.9), underlining the effect of response to chemotherapy. The 2- and 5-year EFS for patients with a rWIR ≥ 2.3 were 85% and 75% versus 55% and 50% for patients with a rWIR < 2.3. Conclusion: The predicted poor chemo response with MRI (rWIR < 2.3) is associated with shorter EFS even when adjusted for known clinical covariates and shows similar results to histological response evaluation. rWIR is a potential tool for future response-based individualized healthcare in osteosarcoma patients before surgical resection.

## 1. Introduction

Osteosarcoma is a malignant bone tumor that generally affects young patients, with a second peak at >40 years of age. Neoadjuvant chemotherapy (NAC) followed by tumor resection is key in curative treatment [1,2,3]. The gold standard for evaluating response to NAC in osteosarcoma patients relies on the histological assessment according to the modified Huvos classification [4,5]. This method has limitations, including the examination of only one slab of tumor tissue, high inter-observer variability, the use of a binary cut-off (≥10% viable tumor cells indicate poor response), and the inability to assess response before resection. These limitations partially cause the lack of clinical implications of histological response assessment in current clinical guidelines as they only become apparent after surgical resection [6]. Consequently, there is a need for a non-invasive prognostic biomarker able to accurately predict chemotherapy treatment response before surgery.

Dynamic contrast-enhanced (DCE-)MRI is an imaging sequence capable of visualizing and quantifying various tumor properties such as tissue perfusion and capillary permeability [7,8]. In a previous study, the relative wash-in rate (rWIR) was described to correlate with a histological response. The rWIR, derived from the baseline imaging’s maximum slope of contrast enhancement (wash-in rate) divided by the post-NAC wash-in rate observed on DCE-MRI time-intensity curves, reflects alterations in tumor perfusion before and after NAC [9]. Utilizing this technique, the response to NAC can be predicted before tumor resection. Association between response assessment before tumor resection and prognosis could thus potentially provide tools for treatment personalization.

Previous studies identified age, tumor size, the presence of metastases at presentation, histological response to chemotherapy, and local recurrence (LR) as risk factors for EFS in osteosarcoma patients treated with curative intent [10,11,12,13,14]. The aim of this multicenter retrospective study is to explore the potential of the rWIR as a prognostic factor for clinical outcome and determine its added value to known prognostic factors.

## 2. Materials and Methods

### 2.1. Design, Setting, and Participants

**Figure 1:**
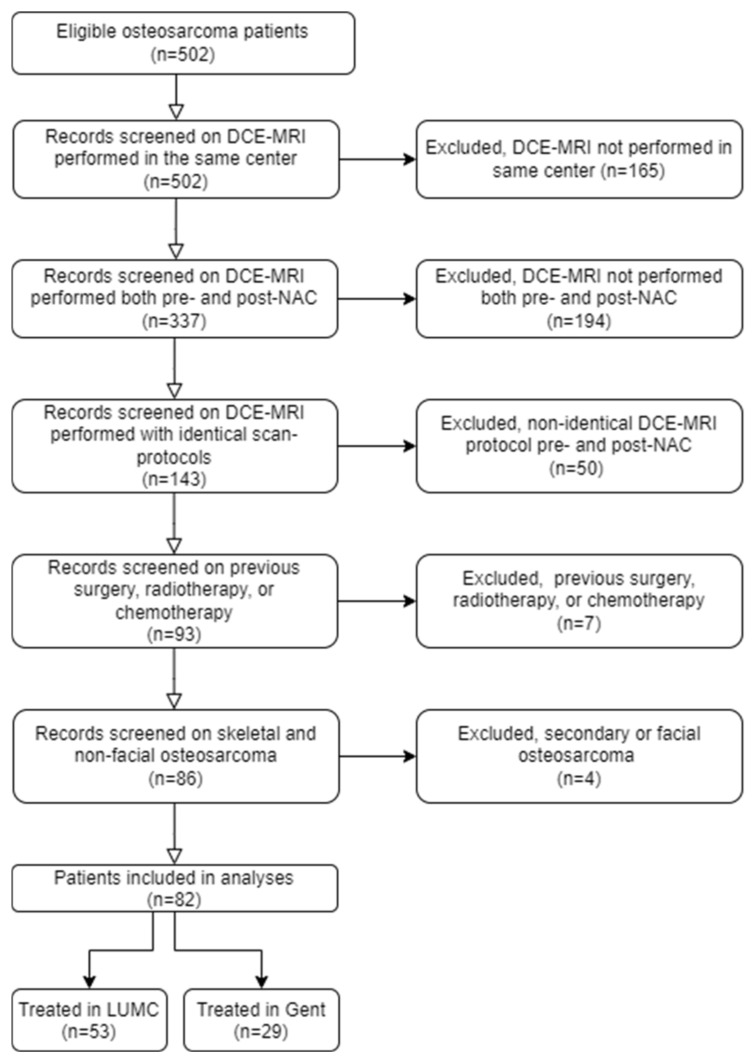
Flowchart of patient selection. DCE-MRI = dynamic contrast-enhanced magnetic resonance imaging; NAC = neoadjuvant chemotherapy.

This multicenter observational retrospective cohort study was conducted between 2005 and 2022 at the Leiden University Medical Center (LUMC) and the Ghent University Hospital (GUH). The study was approved by the ethical review board in both centers, and the need for informed consent was waived due to the retrospective nature of this study (protocol and approval codes: B19.050, BC-09111, and G18.065/SH/gk). Patients with histologically proven skeletal high-grade osteosarcoma and treated with curative intent consisting of chemotherapy and tumor resection were included. All participants underwent DCE-MRI both before and after NAC, using the same scan protocol. Exclusion criteria included patients with secondary osteosarcoma, craniofacial lesions, pre- and post-NAC MRI performed in different centers, a history of previous chemotherapy, and tumor resection or concomitant radiotherapy at the same site. Among 502 initially enrolled patients, 420 were excluded, resulting in a total study population of 82 patients (Figure 1). Of these, 53 were treated at the LUMC and 29 at the GUH. The entire study population of 82 patients was previously studied [9,10]. The first study provided an overview of survival and prognostic factors in 402 patients with skeletal high-grade osteosarcoma, including 53 patients from the current cohort [10]. The second study described the development of the model, predicting the histological response to NAC in 85 osteosarcoma patients, incorporating 82 patients from the current cohort [9]. In this study, we report the potential of the rWIR as a prognostic factor for clinical outcomes and determine its added value to known prognostic factors.

### 2.2. rWIR and Selection of Prognostic Variables

DCE-MRIs, and the corresponding rWIR, were processed in a blinded and independent manner by G.M.K. (4 years of experience) for LUMC patients and T.V.D.B. (4 years of experience) for GUH patients, and rWIR was determined for all included patients. Previous research reported an intraclass correlation coefficient of 0.81 for rWIR in neoadjuvant-treated osteosarcoma, suggesting good repeatability. The rWIR was calculated by dividing the maximum slope of contrast enhancement on the time–intensity curves (wash-in rate) from baseline imaging by the wash-in rate post-NAC. Details can be found in a previous study by Kalisvaart and Van Den Berghe et al. [9]. A prior study by Evenhuis et al. [10] identified prognostic factors for survival in 402 patients treated for a skeletal high-grade osteosarcoma at the LUMC between 1978 and 2017. Age groups, tumor sizes, poor histopathological responses, and metastases at presentation were found to be independent prognostic factors influencing EFS in a multivariate Cox proportional hazard model and were also used in the current analysis [10]. For the current study, patient records were reviewed by the local investigator to obtain demographics, treatment details, and clinical outcomes. Baseline variables included sex, age group (children: 0–<16 years; adolescents and young adults [AYA]: 16–<40 years; older adults: ≥40 years), tumor location, tumor size (≤8 cm or >8 cm), metastases at presentation, histological response according to the Huvos classification (poor response: ≥10% viable tumor cells; good response: <10% viable tumor cells and >90% response) and rWIR [4,9]. The rWIR was analyzed both as a dichotomous variable (rWIR < 2.3 indicated a poor radiological response, and rWIR ≥ 2.3 indicated a good radiological response) and as a continuous variable to evaluate the potential of rWIR to overcome the limitation of the arbitrary threshold for poor response originating from the 10% viable cells threshold in the Huvos classification. EFS was defined as the time from resection to the first event that consisted of LR, new metastases, or tumor-related death. In patients with metastatic disease at presentation, the next consecutive event was considered for EFS.

### 2.3. Follow-Up

Patients were monitored at the outpatient clinic for local control and disease progression. Follow-up protocols varied by center but generally included physical examinations and imaging modalities including computerized tomography (CT), MRI, and radiography.

### 2.4. Statistical Analysis

A multivariate Cox proportional hazard regression model was estimated to study the effect of risk factors on EFS. The model included age group, histological response to chemotherapy, tumor size, and metastases at presentation. A second model included the same prognostic factors but replaced histological response with rWIR. The rWIR was used as a categorical parameter (rWIR < 2.3 indicated a poor radiological response) and afterwards as a continuous parameter. The proportional hazard assumption was tested by using the weighted residuals [15]. This analysis was also conducted on a subpopulation that excluded patients with baseline metastases. Hazard ratios (HRs) and their 95% confidence intervals (CIs) were reported. To evaluate the additional value of rWIR, a comparison between two nested Cox models on the same data set with and without rWIR were compared using a likelihood ratio test [16]. EFS was estimated by employing the Kaplan–Meier (KM) methodology. The median follow-up time was computed using the reversed Kaplan–Meier methodology [17]. The combining batches (ComBat) harmonization method was used to reduce center-specific effects for the rWIR in the previous rWIR study [9]. In the current study, analysis was performed with rWIR after ComBat harmonization and repeated with data without harmonization. Statistical analyses were performed using the SPSS version 25.0 (IBM Corp., Armonk, NY, USA) and R-studio software version 4.2.1. The level of significance was set at a *p*-value of < 0.05.

## 3. Results

### 3.1. Participants and Baseline Characteristics

The cohort consisted of 50 males (61%) and 32 females (39%), with a median age of 17.4 years (interquartile range [IQR]: 13.7–27.7). Respectively, 7/43 patients (16%) with a poor and 5/39 patients (13%) with a good histological response had metastases at presentation (Table 1). The median follow-up time was 11.8 years (95% CI: 11.0–12.7), and there were no dropouts. A total of 33 events were observed during the follow-up. The histological and radiological responses were classified as poor in 43 (52%) and 41 (50%) patients, respectively. In 18 patients (22%), histological and radiological response classifications were discordant. LR was observed in nine patients (11%). All patients with LR underwent re-resection, without additional chemo- or radiotherapy. The development of metastases during follow-up was observed in 31 patients (38%) that were treated with metastasectomy alone (n = 16, 52%), metastasectomy and chemotherapy (n = 5, 17%), metastasectomy and radiotherapy (n = 3, 10%), chemotherapy (n = 3, 10%), and metastasectomy, chemotherapy, and radiotherapy (n = 1, 3%), and 3 patients (10%) were not treated due to poor prognosis.

### 3.2. Prognostic Factors’ Effect on EFS

In the first model (histological response included), none of the variables were significantly associated with EFS. Histological response (HR: 1.8; 95% CI: 0.9–3.8; reference category: good responder) and metastases at presentation (HR: 2.3; 95% CI: 0.9–5.8; reference category: no metastases) were the most influential factors, though not significant (Table 2). In the second model, the rWIR was used instead of the histological response to chemotherapy. The rWIR < 2.3 was significantly associated with worse EFS (HR: 2.4; 95% CI: 1.1–5.0; reference category: rWIR > 2.3), but metastases at presentation (HR: 2.3; 95% CI: 0.9–5.9) was not. Repeating the analysis in the none ComBat harmonization cohort showed that rWIR < 2.3 was still associated with EFS (HR: 2.8; 95% CI: 1.3–5.9). In the third model, the rWIR as a continuous variable was incorporated. None of the included variables were significantly associated with EFS, although the rWIR as a continuous variable (HR 0.8, 95% CI 0.6–1.0) and metastases at presentation (HR 1.9, 95% CI 0.7–4.9) were the most influential variables. A further subpopulation analysis, including 70 patients (85%) without metastases at presentation, revealed that rWIR as a binary variable nearly approached significance, suggesting an association with EFS (HR: 2.3; 95% CI: 1.0–5.2) (Table 3). rWIR as a continuous variable was significantly associated with EFS (HR: 0.7; 95% CI: 0.5–0.9). Thus, an increase in the continuous rWIR (and a decrease in wash-in rate during NAC) resulted in an increased EFS. The proportional hazard assumption for each covariate was not violated. Analysis of deviance suggests that adding rWIR to the multivariate Cox model leads to an improved fit over the model (*p* = 0.07), although further research is necessary to obtain more robust results.

### 3.3. Event-Free Survival

**Figure 2:**
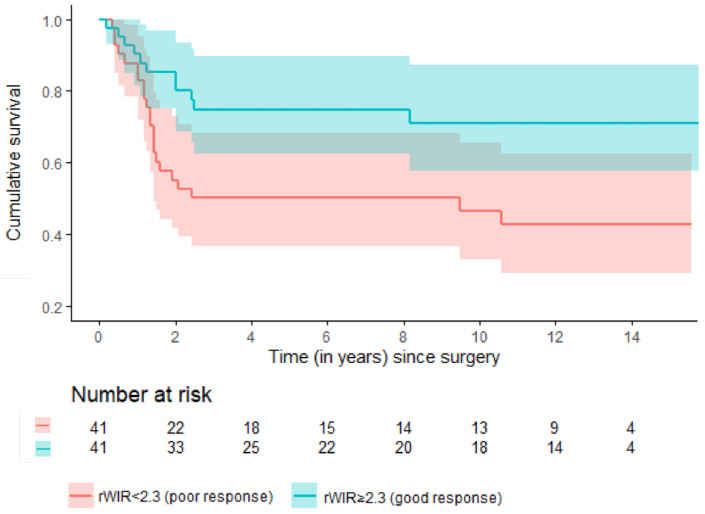
Estimated event-free survival among good and poor responders based on the rWIR with a cut-off of 2.3.

The 2- and 5-year EFS for patients with a rWIR of ≥2.3 (a good response) were 85% (95% CI: 74–96) and 75% (95% CI: 62–89) versus 55% (95% CI: 40–70) and 50% (95% CI: 35–66) for patients with a rWIR of <2.3 (a poor response) (Figure 2).

**Figure 3:**
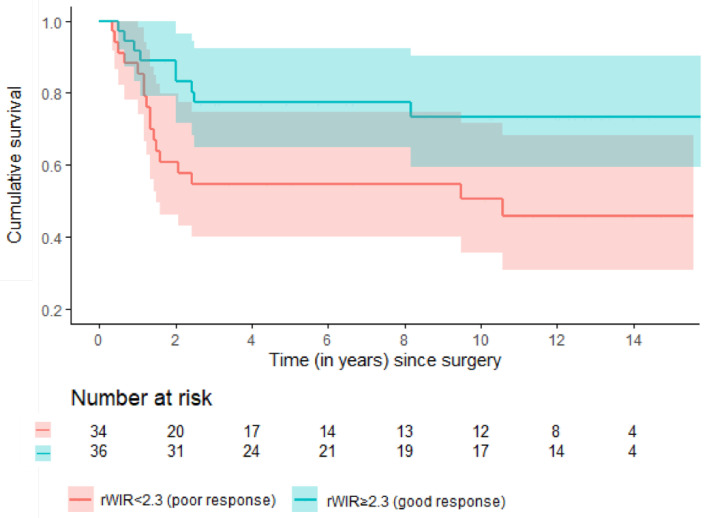
Estimated event-free survival among good and poor responders based on the rWIR with a cut-off of 2.3 in a subpopulation of 70 patients without metastases at presentation.

Among 70 patients without metastases at presentation, 24 (35%) experienced an event. The 2- and 5-year EFS for patients with a rWIR of ≥ 2.3 (a good response) were 89% (95% CI: 79–99) and 77% (95% CI: 63–91) versus 61% (95% CI: 44–78) and 55% (95% CI: 38–72) for patients with a rWIR of <2.3 (a poor response) (Figure 3). The 2- and 5-year recurrence-free survival for patients with a rWIR of ≥2.3 were 98% (95% CI: 93–100) and 98% (95% CI: 93–100) versus 87% (95% CI: 77–97) and 82% (95% CI: 69–94) for patients with a rWIR of <2.3. The 2- and 5-year metastasis-free survival for patients with a rWIR ≥ of 2.3 were 85% (95% CI: 75–96) and 75% (95% CI: 61–88) versus 57% (95% CI: 41–72) and 52% (95% CI: 36–67) for patients with a rWIR of <2.3.

## 4. Discussion

In this multicenter study, rWIR, as determined using pre- and post-NAC DCE-MRI, was found to be associated with EFS in patients with osteosarcoma. To the best of our knowledge, this is the first study to date on the prognostic value of rWIR for EFS. Results show that a previously determined cut-off of rWIR < 2.3 is associated with poor EFS when adjusted for age group, tumor size, and metastases at presentation. Furthermore, the continuous rWIR was significantly associated with EFS in a subpopulation without metastases at presentation. Our findings suggest rWIR might have value for response stratification in patients without metastases at diagnosis, as rWIR can be determined before resection and used as a continuous variable in survival prediction. This contrasts with the traditionally used histological response, and in our population, it was not significantly associated with EFS.

The link between DCE-MRI-based perfusion characteristics and histological responses has been described extensively in the literature [7,18]. Guo et al. and Hao et al. found that several features, describing tissue permeability and perfusion, were correlated with histologic responses [7,19]. In a previous rWIR study, standardized optimal methods to use perfusion characteristics, specifically the derived time–intensity curve, for histological response prediction were identified [9]. This determination of the association with biological changes in tumor tissues allows a more explainable association of this imaging characteristic with survival outcomes in the current study.

Although in this study the use of pre- and post-NAC DCE-MRI was investigated to predict clinical outcome of osteosarcoma patients, there are other functional imaging techniques such as PET-CT and DWI that have been described for predicting the response to chemotherapy [7,20,21]. These studies typically predict the histological response instead of survival. Studies evaluating quantitative, texture-based imaging features (radiomics) for predicting treatment response and survival have not yet resulted in widely accepted and implemented prognostic imaging biomarkers [22,23,24,25,26]. Interpretation of results in radiomics studies is often complicated by a lack of correcting methods for other prognostic factors, such as the presence of metastases or age and complexity of radiomics models. Furthermore, implementation is challenging, since specific software, quality control, and adherence to standardized study protocols are needed to assure reliable results and reproducibility of the results [27,28]. In this regard, the rWIR is a practical and explainable biomarker that is associated with clinical outcome when adjusted for most important covariates. Moreover, it facilitates a deeper understanding of tumor behavior during treatment, providing radiologists and oncologists with a comprehensive tool for analysis and interpretation.

A limitation of this study is that it included patients previously described in the study deciphering the association between rWIR and histological response. Ideally, new patients should have been included. However, the model, which was used to identify rWIR, was not trained using survival data, causing the current study to provide valuable added information on patient stratification. Additionally, T1-mapping was not available in this study, preventing the use of Tofts features from the analyses [29]. DCE-MRI features based on the Tofts-model, such as differences in relative extravascular extracellular space and influx volume transfer constant, have been shown to be prognostic factors for EFS and overall survival (OS) [7,19]. However, limited cohort sizes, arbitrary cut-offs for radiological response classification, and a lack of correction for other prognostic factors limit the interpretability of these results described in the literature. Nevertheless, future studies should determine if Tofts modeling strengthens the prognostic value of DCE-MRI characteristics for patient stratification.

The rWIR holds potential for the early evaluation of NAC treatment response and EFS prediction in a non-invasive way before tumor resection. Moreover, patients with a poor radiological response to NAC experienced recurrences more often and seemed to have a shorter recurrence-free survival. Once further investigated and validated in larger sample sizes, this method could allow for standardized response monitoring in studies on neoadjuvant therapies, for example, in the development of new chemotherapy regimens. This, in turn, might contribute to individualized therapies and decision making. It involves the potential to either avoid or intensify ineffective chemotherapy cycles. Additionally, it might justify a more invasive surgical or adjuvant radiotherapy treatment in patients with predicted poor prognosis, aiming to prevent local recurrences and improve survival outcomes.

## 5. Conclusions

The rWIR is associated with EFS and is a valuable addition to other clinical parameters. Future prospective studies on response monitoring and EFS prediction should be compared to the performance of rWIR.

## Figures and Tables

**Table 1:** Characteristics of the overall cohort.

Characteristics	N Total (%)	LUMC	GUH	*p*-Value
**Total**	82	53 (65)	29 (35)	
**Sex**				0.20
Male	50 (61)	35 (66)	15 (52)	
Female	32 (39)	18 (34)	14 (48)	
**Age group**				0.16
Children (0–<16 yrs)	35 (43)	19 (36)	16 (55)	
AYA (16–<40 yrs)	35 (43)	24 (45)	11 (38)	
Older adults (≥40 yrs)	12 (15)	10 (19)	2 (7)	
**Location tumor**	86			0.10
Lower extremity	68 (83)	41 (77)	27 (93)	
Upper extremity	7 (9)	5 (9)	2 (7)	
Axial skeleton	7 (9)	7 (13)	0	
**Tumor size**				0.92
Small (≤8 cm)	39 (48)	25 (47)	14 (48)	
Large (>8 cm)	43 (52)	28 (53)	15 (52)	
**Metastases at presentation**				0.98
No	70 (85)	46 (87)	24 (83)	
Yes	12 (12)	7 (13)	5 (17)	
**Preoperative CTx treatment**	81	52	29	0.17
1 MAP or 2 AP completed	4 (5)	4 (8)	0 (0)	
2 MAP or 3 AP completed	71 (87)	43 (83)	28 (97)	
>2 MAP or >3 AP completed	6 (7)	5 (10)	1 (3)	
**Histological response to CTx**				0.20
Poor (≥10% viable tumor cells)	43 (52)	25 (47)	18 (62)	
Good (<10% viable tumor cells)	39 (39)	28 (53)	11 (38)	
**Radiological response to CTx**				0.49
Poor response (rWIR < 2.3)	41 (50)	28 (53)	13 (45)	
Good response (rWIR ≥ 2.3)	41 (50)	25 (47)	16 (55)	
**Local recurrence**				0.38
No	73 (89)	46 (78)	27 (93)	
Yes	9 (11)	7 (13)	2 (7)	
**Metastases during follow-up**				0.06
No	51 (62)	29 (55)	22 (76)	
Yes	31 (38)	24 (45)	7 (24)	

AYA = adolescents and young adults; yrs = years; MAP = Methotrexate, Adriamycin, and Cisplatin; AP = Adriamycin and Cisplatin; CTx = chemotherapy; and rWIR = relative wash-in rate.

**Table 2:** Hazard ratios (HRs) along with the 95% confidence intervals using multivariate Cox regression models for EFS in a study population (*n* = 82), with prognostic factors including histological response (left), rWIR as a binary variable (middle), and rWIR as a continuous variable (right) as the prognostic factors.

Factors	HR	95% CI	Factors	HR	95% CI	Factors	HR	95% CI
**Age Group**			**Age group**			**Age Group**		
Children	Ref		Children	Ref		Children	Ref	
AYA	1.36	0.61–3.03	AYA	1.43	0.64–3.22	AYA	1.32	0.59–2.98
Older adults	1.26	0.45–3.55	Older adults	1.55	0.55–4.41	Older adults	1.41	0.50–3.97
**Tumour size**			**Tumour size**			**Tumour size**		
Small ≤ 8 cm	Ref		Small ≤ 8 cm	Ref		Small ≤ 8 cm	Ref	
Large > 8 cm	0.90	0.46–2.00	Large > 8 cm	0.97	0.47–2.00	Large > 8 cm	0.96	0.46–2.01
**Histological response to CTx**			**DCE-MRI response (binary) to CTx**			**DCE-MRI response**		
Good response (<10% viable tum. cells)	Ref		Good response (rWIR ≥ 2.3)	Ref		**(continuous) to CTx**	0.78	0.60–1.01
Poor response (≥10% viable tum. cells)	1.82	0.86–3.84	Poor response (rWIR < 2.3)	2.39	1.14–5.01			
**Metastases at presentation**			**Metastases at presentation**			**Metastases at presentation**		
No	Ref		No	Ref		No	Ref	
Yes	2.29	0.90–5.83	Yes	2.31	0.90–5.92	Yes	1.85	0.70–4.94

HR = hazard ratio; ref = reference category; 95% CI = 95% confidence interval; CTx = chemotherapy; DCE-MRI = dynamic contrast-enhanced magnetic resonance imaging; AYA = adolescents and young adults; and rWIR = relative wash-in rate.

**Table 3:** Hazard ratios (HRs) along with the 95% confidence intervals using multivariate Cox regression models for EFS with prognostic factors including histological response (left), rWIR as a binary variable (middle), and rWIR as a continuous variable (right) as the prognostic factors in a subpopulation of 70 patients without metastases at presentation.

Factors	HR	95% CI	Factors	HR	95% CI	Factors	HR	95% CI
**Age group**			**Age group**			**Age group**		
Children	Ref		Children	Ref		Children	Ref	
AYA	1.43	0.59–3.46	AYA	1.46	0.61–3.53	AYA	1.28	0.53–3.13
Older adults	2.11	0.66–6.79	Older adults	2.30	0.74–7.21	Older adults	2.02	0.63–6.50
**Tumour size**			**Tumour size**			**Tumour size**		
Small ≤ 8 cm	Ref		Small ≤ 8 cm	Ref		Small ≤ 8 cm	Ref	
Large > 8 cm	1.26	0.55–2.92	Large > 8 cm	1.33	0.60–2.97	Large > 8 cm	1.23	0.54–2.80
**Histological response to CTx**			**DCE-MRI response (binary) to CTx**			**DCE-MRI response (continuous) to**		
Good responder (<10% viable cells)	Ref		Good responder (rWIR ≥ 2.3)	Ref		**CTx**	0.69	0.50–0.94
Poor responder (≥10% viable cells)	1.98	0.84–4.67	Poor responder (rWIR < 2.3)	2.28	1.00–5.19			

HR = hazard ratio; ref = reference category; 95% CI = 95% confidence interval; CTx = chemotherapy; DCE-MRI = dynamic contrast-enhanced magnetic resonance imaging; AYA = adolescents and young adults; and rWIR = relative wash-in rate.

## Data Availability

The (anonymized) data presented in this study are available on request from the corresponding author due to privacy reasons.

## Abbreviations

DCE-MRI = dynamic contrast-enhanced magnetic resonance imaging; rWIR = relative wash-in rate; CT = computerized tomography; EFS = event-free survival; OS = overall survival; NAC = neoadjuvant chemotherapy; and ComBat = combining batches.
