# Correction: Kalisvaart et al. Relative Wash-In Rate in Dynamic Contrast-Enhanced Magnetic Resonance Imaging as a New Prognostic Biomarker for Event-Free Survival in 82 Patients with Osteosarcoma: A Multicenter Study. *Cancers* 2024, *16*, 1954

**DOI:** 10.3390/cancers17162603

**Published:** 2025-08-08

**Authors:** Gijsbert M. Kalisvaart, Richard E. Evenhuis, Willem Grootjans, Thomas Van Den Berghe, Martijn Callens, Judith V. M. G. Bovée, David Creytens, Hans Gelderblom, Frank M. Speetjens, Lore Lapeire, Gwen Sys, Marta Fiocco, Koenraad L. Verstraete, Michiel A. J. van de Sande, Johan L. Bloem

**Affiliations:** 1 Department of Radiology, Leiden University Medical Center, 2333 Leiden, The Netherlands; g.m.kalisvaart@lumc.nl (G.M.K.); j.l.bloem@lumc.nl (J.L.B.); 2 Department of Orthopedic Surgery, Leiden University Medical Center, Albinusdreef 2, 2333 Leiden, The Netherlands; 3 Department of Radiology, Ghent University Hospital, 9000 Ghent, Belgium; thovdnbe.vandenberghe@ugent.be (T.V.D.B.);; 4 Department of Pathology, Leiden University Medical Center, 2333 Leiden, The Netherlands; 5 Department of Pathology, Ghent University Hospital, 9000 Ghent, Belgium; david.creytens@uzgent.be; 6 Department of Medical Oncology, Leiden University Medical Center, 2333 Leiden, The Netherlands; 7 Department of Medical Oncology, Ghent University Hospital, 9000 Ghent, Belgium; lore.lapeire@uzgent.be; 8 Department of Orthopedic Surgery and Traumatology, Ghent University Hospital, 9000 Ghent, Belgium; gwen.sys@uzgent.be; 9 Department of Biomedical Science, Section Medical Statistics, Leiden University Medical Center, 2333 Leiden, The Netherlands; 10 Center for Pediatric Oncology, Princess Maxima Center, 3584 Utrecht, The Netherlands; 11 Mathematical Institute, Leiden University, 2300 Leiden, The Netherlands

## Error in Figure 2

In the original publication [1], there was a mistake in the legend of Figure 2 as published. In the original version the legend wrongly states the red line accounts for patients with ‘rWIR ≥ 2.3 (good response),’ whereas it actually accounts for patients with poor response. The corrected Figure 2 appears below. 

## Error in Figure 3

In the original publication, there was a mistake in the legend of Figure 3 as published. In the original version the legend wrongly states the red line accounts for patients with ‘rWIR ≥ 2.3 (good response),’ whereas it actually accounts for patients with poor response. The corrected Figure 3 appears below. 

## Error in Table 2

In the original publication, there was a mistake in Table 2 as published. The fourth column of the original table wrongly defines good and poor radiological responders. rWIR < 2.3 should define poor response and rWIR ≥ 2.3 should define good response. The corrected Table 2 appears below. 

## Error in Table 3

In the original publication, there was a mistake in Table 3 as published. The fourth column of the original table wrongly defines good and poor radiological responders. rWIR < 2.3 should define poor response and rWIR ≥ 2.3 should define good response. The corrected Table 3 appears below. 

The authors state that the scientific conclusions are unaffected. This correction was approved by the Academic Editor. The original publication has also been updated.

## Figures and Tables

**Figure 2:**
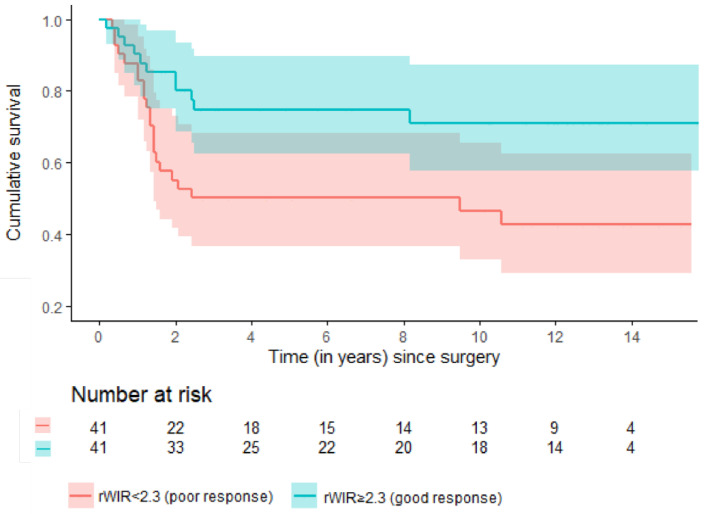
Estimated event-free survival among good and poor responders based on the rWIR with a cut-off of 2.3.

**Figure 3:**
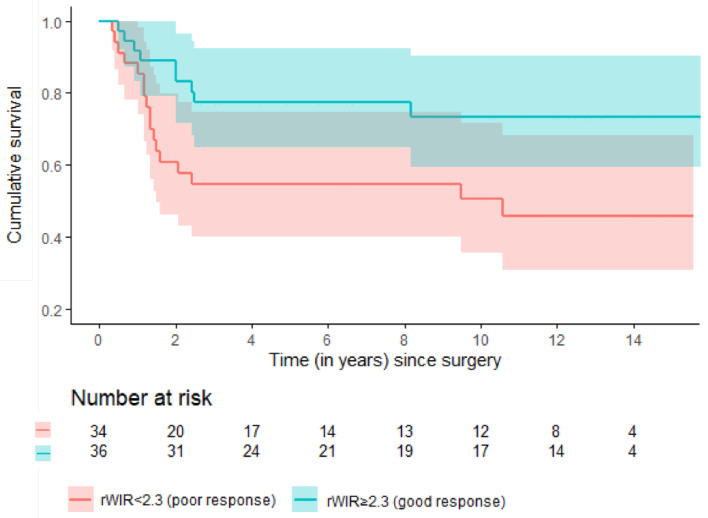
Estimated event-free survival among good and poor responders based on the rWIR with a cut-off of 2.3 in a subpopulation of 70 patients without metastases at presentation.

**Table 2:** Hazard ratios (HRs) along with the 95% confidence intervals using multivariate Cox regression models for EFS in a study population (*n* = 82), with prognostic factors including histological response (left), rWIR as a binary variable (middle), and rWIR as a continuous variable (right) as the prognostic factors.

Factors	HR	95% CI	Factors	HR	95% CI	Factors	HR	95% CI
**Age Group**			**Age group**			**Age Group**		
Children	Ref		Children	Ref		Children	Ref	
AYA	1.36	0.61–3.03	AYA	1.43	0.64–3.22	AYA	1.32	0.59–2.98
Older adults	1.26	0.45–3.55	Older adults	1.55	0.55–4.41	Older adults	1.41	0.50–3.97
**Tumour size**			**Tumour size**			**Tumour size**		
Small ≤ 8 cm	Ref		Small ≤ 8 cm	Ref		Small ≤ 8 cm	Ref	
Large > 8 cm	0.90	0.46–2.00	Large > 8 cm	0.97	0.47–2.00	Large > 8 cm	0.96	0.46–2.01
**Histological response to CTx**			**DCE-MRI response (binary) to CTx**			**DCE-MRI response**		
Good response (<10% viable tum. cells)	Ref		Good response (rWIR ≥ 2.3)	Ref		**(continuous) to CTx**	0.78	0.60–1.01
Poor response (≥10% viable tum. cells)	1.82	0.86–3.84	Poor response (rWIR < 2.3)	2.39	1.14–5.01			
**Metastases at presentation**			**Metastases at presentation**			**Metastases at presentation**		
No	Ref		No	Ref		No	Ref	
Yes	2.29	0.90–5.83	Yes	2.31	0.90–5.92	Yes	1.85	0.70–4.94

HR = hazard ratio; ref = reference category; 95% CI = 95% confidence interval; CTx = chemotherapy; DCE-MRI = dynamic contrast-enhanced magnetic resonance imaging; AYA = adolescents and young adults; and rWIR = relative wash-in rate.

**Table 3:** Hazard ratios (HRs) along with the 95% confidence intervals using multivariate Cox regression models for EFS with prognostic factors including histological response (left), rWIR as a binary variable (middle), and rWIR as a continuous variable (right) as the prognostic factors in a subpopulation of 70 patients without metastases at presentation.

Factors	HR	95% CI	Factors	HR	95% CI	Factors	HR	95% CI
**Age group**			**Age group**			**Age group**		
Children	Ref		Children	Ref		Children	Ref	
AYA	1.43	0.59–3.46	AYA	1.46	0.61–3.53	AYA	1.28	0.53–3.13
Older adults	2.11	0.66–6.79	Older adults	2.30	0.74–7.21	Older adults	2.02	0.63–6.50
**Tumour size**			**Tumour size**			**Tumour size**		
Small ≤ 8 cm	Ref		Small ≤ 8 cm	Ref		Small ≤ 8 cm	Ref	
Large > 8 cm	1.26	0.55–2.92	Large > 8 cm	1.33	0.60–2.97	Large > 8 cm	1.23	0.54–2.80
**Histological response to CTx**			**DCE-MRI response (binary) to CTx**			**DCE-MRI response (continuous) to**		
Good responder (<10% viable cells)	Ref		Good responder (rWIR ≥ 2.3)	Ref		**CTx**	0.69	0.50–0.94
Poor responder (≥10% viable cells)	1.98	0.84–4.67	Poor responder (rWIR < 2.3)	2.28	1.00–5.19			

HR = hazard ratio; ref = reference category; 95% CI = 95% confidence interval; CTx = chemotherapy; DCE-MRI = dynamic contrast-enhanced magnetic resonance imaging; AYA = adolescents and young adults; and rWIR = relative wash-in rate.
